# Diabetes and oral health: A comparative cross-sectional analysis of DMFT index among diabetic, pre-diabetic, and non-diabetic adults

**DOI:** 10.1371/journal.pone.0331775

**Published:** 2025-09-25

**Authors:** Mohammad Samami, Amir Valaei-Barhagh, Farahnaz Joukar, Soheil Hassanipour, Maryam Rabiei, Arman Habibi, Mohammad Reza Naghipour, Fariborz Mansour-Ghanaei

**Affiliations:** 1 Dental Sciences Research Center, Department of Oral and Maxillofacial Medicine, School of Dentistry, Guilan University of Medical Sciences, Rasht, Iran; 2 Gastrointestinal and Liver Diseases Research Center, Guilan University of Medical Sciences, Rasht, Iran; 3 Student Research Committee, School of Medicine, Guilan University of Medical Sciences, Rasht, Iran; Shahid Beheshti University of Medical Sciences School of Dentistry, IRAN, ISLAMIC REPUBLIC OF

## Abstract

**Objective:**

The objective of this study was to compare the DMFT index (decayed, missing, and filled teeth) between diabetic, pre-diabetic, and non-diabetic subjects and to determine whether sociodemographic (including age, education, or socioeconomic status) or health-related factors (such as BMI, smoking, or physical activity) were associated with DMFT levels in people with diabetes.

**Methods:**

This study is cross-sectional and part of the PERSIAN Guilan cohort study. Demographic information, body mass index (BMI), cigarettes and hookah, alcohol and drug use, co-morbidity diseases, socio-economic status (SES), and DMFT of all 35–70-year-olds were investigated. Classification of diabetes status was done based on the result of the FBS test or self-report of the participant, or the use of hypoglycemic drugs.

**Results:**

Out of 10520 people who participated in the study, 2531 people had diabetes, 1837 people had pre-diabetes, and 6152 people were non-diabetics. The average DMFT in diabetic, pre-diabetic, and non-diabetic participants was 16.03, 14.63, and 13.94, respectively, and the differences in DMFT between the three groups were statistically significant (P < 0.05). The risk for higher DMFT was older patient age, lower educational status, lower BMI, less physical activity, smoking, alcohol consumption, and not brushing. However, drug use is considered a risk factor only for diabetics.

**Conclusion:**

In all groups, higher DMFT risk factors included older age, lower education, reduced BMI, less physical activity, smoking, alcohol consumption, and inadequate teeth brushing. Notably, drug use is regarded as a risk factor exclusively among participants with diabetes.

## Introduction

Diabetes mellitus (DM) is a major metabolic and multifactorial disorder determined by chronic hyperglycemia due to insulin dysfunction, insulin secretion disorder, or both [1]. This condition has emerged as a silent epidemic with alarming global implications [1,2]. According to the International Diabetes Federation (IDF) estimated that approximately 537 million adults worldwide had diabetes in 2021, representing one in ten adults aged 20–79 years. These numbers are projected to increase dramatically, reaching 643 million by 2030 and 783 million by 2045 [3]. The rising prevalence of diabetes has transformed it from the twentieth leading cause of death in 1990 to the fifth leading cause by 2019, highlighting its growing impact on global mortality [4]. Based on the Prospective Epidemiological Research Studies in IrAN (PERSIAN) cohort study, 15% and 25.4% of 163770 participants had diabetes and pre-diabetes, respectively, between 2014 and 2020 [5]. Current estimates suggest that 5.5 million Iranians were affected by diabetes in 2021, with projections indicating this number will nearly double to 9.2 million by 2030 [2,3,6].

The chronic hyperglycemia associated with diabetes leads to widespread complications affecting multiple organ systems throughout the body. Diabetes significantly increases the risk of microvascular complications, including nephropathy, retinopathy, and neuropathy, as well as macrovascular complications such as cardiovascular disease and stroke [7,8]. These complications arise through complex pathophysiological mechanisms involving oxidative stress, inflammation, and endothelial dysfunction [9,10] Among the various complications, diabetes also has profound effects on oral health, manifesting as dry mouth, periodontitis, gingivitis, increased dental caries, delayed wound healing, salivary gland dysfunction, and heightened susceptibility to opportunistic infections such as oral candidiasis [8]. The bidirectional relationship between diabetes and oral health is particularly significant, as poor oral hygiene can worsen glycemic control while diabetes-related complications can further deteriorate oral health status [11].

Oral health is essential to public health, directly associated with people’s health [12]. It is represented by some indicators such as the Decayed, Missing, and Filled Teeth (DMFT), the Decayed, Missing, and Filled Surfaces (DMFS), the Community Index of Periodontal Treatment Needs (CPITN), Oral Hygiene Index (OHI), among others. [13–15]. The DMFT index serves as a valuable tool for monitoring oral health trends, comparing different populations, and assessing the effectiveness of preventive interventions [12,14]. Given the established link between diabetes and oral health complications, and the utility of DMFT as an indicator of dental caries experience. this study aims to compare DMFT scores between diabetic, pre-diabetic, and non-diabetic participants in the PERSIAN Guilan Cohort Study (PGCS). Additionally, we seek to determine whether sociodemographic factors or health-related factors are associated with DMFT levels among individuals with diabetes.

## Materials and methods

### Study design and setting

This study is a cross-sectional analysis of data from PGCS participants. Between October 8, 2014, and January 20, 2017, 10520 men and women aged 35–70 years were included in the PERSIAN cohort in Guilan Province, Northern Iran. The study’s data were accessed on February 15, 2025, following the approval of the research by the ethics committee at Guilan University.

The PECO framework guided the study:

P (Population): Adults (typically defined as ≥18 years).

E (Exposure): Diabetes status (specifically categorized as: Diabetic, Pre-Diabetic).

C (Comparison): Non-diabetic adults.

O (Outcome): DMFT Index (Decayed, Missing, Filled Teeth index).

The inclusion criteria for this study mandated enrollment in the PGCS [16] and the availability of DMFT index assessments. As a result, five participants from the PGCS cohort were excluded from the final analysis because their DMFT records were incomplete or undocumented.

The study had specific exclusion criteria to ensure a focused participant group. Individuals who were unable to attend the clinic for a physical examination, those with intellectual disabilities, and individuals unwilling to participate in the research were not included in the study [16].

Qualified subjects were contacted through mobile phones by educated interviewers who could communicate in the native language of this region with participants in the study. After signing informed consent, data, including demographic, clinical, and laboratory characteristics, were recorded by educated research colleagues [17]. Two educated, skilled examiners recorded DMFT and other oral health data [18] with inter-rater reliability evaluated using Cohen’s kappa (κ = 0.608, 95% CI: 0.54–0.68). Discrepancies were resolved through education and supervision by a dentist, according to the World Health Organization (WHO) Oral Health Surveys Basic Methods [19].

### Statement of ethics

This study has been approved by the Ethics Committee of Guilan University of Medical Sciences (IR.GUMS.REC.1401.571). We confirm that all methods were performed following the relevant guidelines and regulations. At the time of enrollment, written informed consent to participate in the study was obtained from participants (or their legal guardians in the case of illiterate participants). The aim and steps of the study were completely explained to the participants, and then anyone who filled out the informed consent was included. They were free to leave the study at any time and for any reason. The funders had no role in the study design, data collection and analysis, decision to publish, or preparation of the manuscript.

### Measurements and procedures

Participants were divided into 3 groups: diabetic, pre-diabetic, and non-diabetic people. Participants with DM were defined as having a history of anti-diabetic medicine consumption, having a history of detected DM, or having fasting blood sugar (FBS) > 126 mg/dl in the primary cohort laboratory data [16]. Prediabetes is defined by a FBS level ranging from 100 to 125 mg/dL in participants who do not have a diagnosis of diabetes. [20] The remaining participants were classified as members of the non-diabetic group.

To evaluate the participants’ oral health condition, the most important outcome of this study, DMFT, a discrete variable, was used. The DMFT score was calculated as the number of decayed teeth (D), missed teeth (M), and filled teeth (F). The mean DMFT for all samples is measured by dividing the sum of all DMFT scores by the total number of participants.

The following variables, such as gender (male or female), level of education (illiterate, 1–5 years of education, 6–12 years of education, or university), physical activity (low, moderate, high), age (35–44, 45–55, or >55), The Socioeconomic status (SES) of participants (low, moderate, or high), Smoking (smoker, non-smoker) and number of cigarettes per day, Using hookah (yes/no), drug use (yes/no), residency (Urban/Rural), using alcohol (yes/no), BMI (<18.5, 18.5–24.9, 25–29.9, > 30), using mouthwash (yes/no), flossing (yes/no), brushing teeth (no brushing, once daily, twice daily, three times a day, others), and co-morbidity diseases (no diseases, one disease, or two diseases or more) were analyzed in diabetic, pre-diabetic, and non-diabetic people to conclude that which of these factors were risk factors [16].

Edentulism was operationally defined as the presence of complete dentures. Participants fitted with complete dentures were identified and subsequently excluded from sensitivity analyses to reduce potential bias arising from inflated ‘Missing’ (M) values within the DMFT index. Additionally, one participant did not complete the questionnaire assessing the use of hookah, alcohol, and illicit substances.

Physical activity in the PERSIAN cohort was gauged through participants’ self-reported daily activities using Metabolic equivalents (METs) derived from a questionnaire.

A MET is defined as the resting metabolic rate, which is approximately 3.5 ml of oxygen per kilogram per minute. As four METs require 16 mL 02/kg/min. MET values for each activity were obtained using a compendium of physical activities [21].

The SES of participants was evaluated using a constructed household wealth indicator. The participants were questioned about whether they owned certain durable assets such as a PC/laptop, CD/DVD player, smartphone, refrigerator, freezer, dishwashing machine, 3D TV, car, vacuum cleaner, sewing machine, cooler, microwave, motorcycle, per capita rooms, and the type of ownership of the home. Participants were also questioned about infrastructure facilities (internet access, piped drinking water access). Based on what was mentioned above, we created the wealth index utilizing the principal component analysis (PCA) technique [22,23].

Co-morbidity diseases were defined as one of the following: ischemic heart disease, history of MI (myocardial infarction), history of stroke, kidney failure, fatty liver, hepatitis B or C, chronic lung diseases, thyroid diseases, kidney stones or gall bladder, rheumatism diseases, chronic headaches, and epilepsy [16].

### Statistical analysis

The normality of variables was assessed through the Kolmogorov-Smirnov test. The one-way ANOVA examined differences in mean DMFT indices across diabetic status groups (diabetic, prediabetic, non-diabetic). To evaluate pairwise differences between the groups, we conducted Tukey’s post hoc analysis. Three two-way ANOVA models (Type III sum of squares) were employed to evaluate Interaction effects between diabetic status and each secondary factor. When comparing two quantitative variables (number of cigarettes and DMFT), we applied the Pearson correlation. To identify the risk factors that affect changes in DMFT, we used multiple linear regression. All analyses were performed using the IBM SPSS Statistics version 27 software. The significance level was considered to be less than 0.05 for all tests.

## Results

Of the 10520 subjects who participated in the study, 5633 (53.5%) were female. The mean age of the study population was 51.51 ± 8.8 years, and 5907 (56.1%) were rural residents. Table 1 presents the Baseline Characteristics of the PERSIAN Guilan cohort study.

**Table 1:** Baseline characteristics of the PERSIAN Guilan Cohort Study (PGCS) participants.

Variables	Classifications	N	%
Age (years)	35-45	3139	29.8%
	45-55	3854	36.6%
	>55	3527	33.6%
Gender	Male	4887	46.5%
	Female	5633	53.5%
Residency	Urban	4613	43.84%
	Rural	5907	56.16%
Level of Education	illiterate	1738	16.5%
	Primary	3312	31.5%
	Secondary	4832	45.9%
	Higher	638	6.1%
BMI	<18.5	141	1.3%
	≥18.5 ≤ 25	2746	26.1%
	>25 < 30	4198	39.9%
	>30	3435	32.7%
Socioeconomic status	Low	3495	33.2%
	Moderate	3505	33.3%
	High	3515	33.4%
Smoking habits	Yes	2584	24.6%
	No	7936	75.4%
Using Alcohol	Yes	1395	13.3%
	No	9125	86.7%

**Fig 1:**
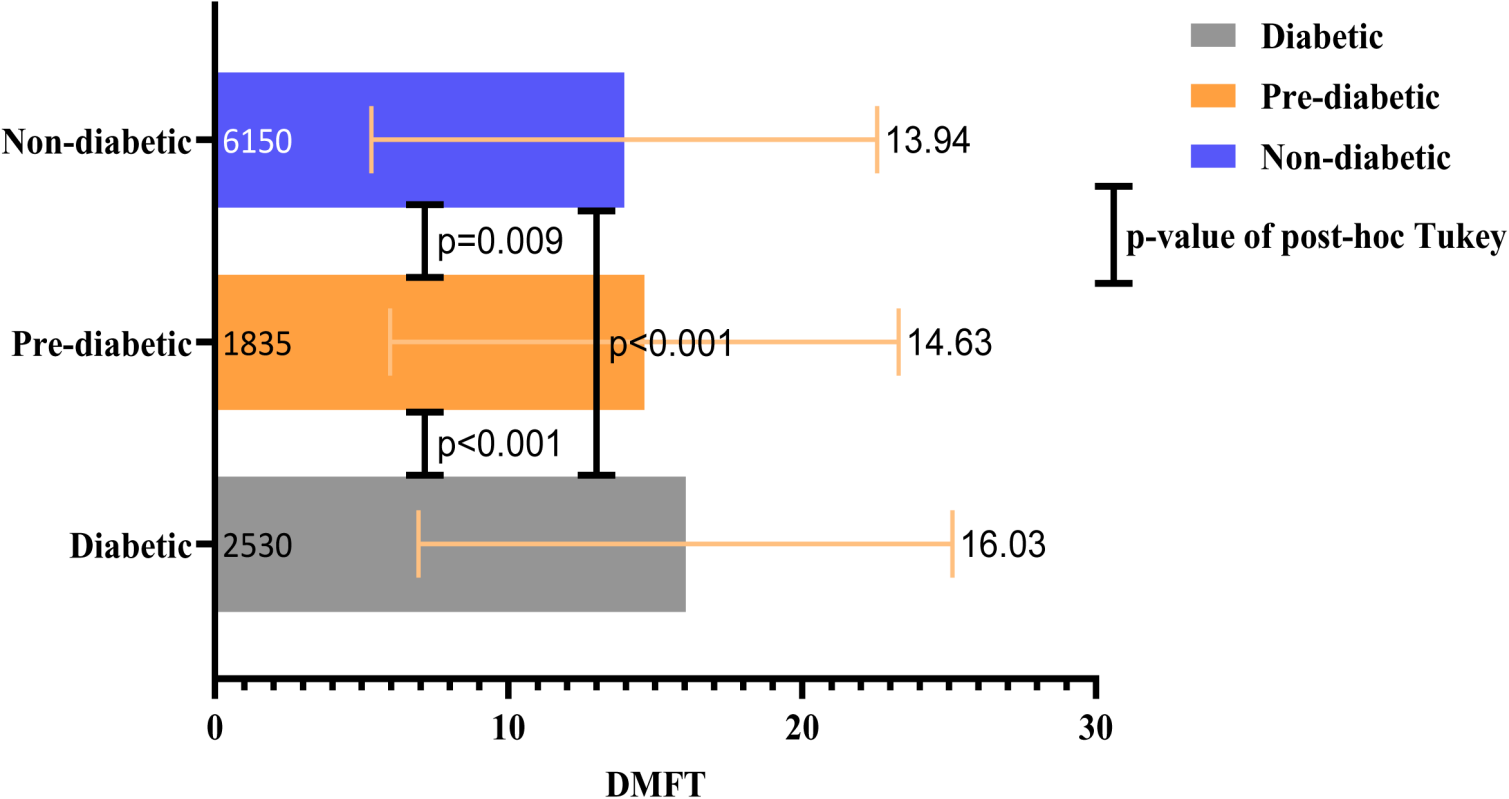
Bar chart indicating mean DMFT  ± SD and the results of post-hoc Tukey.

**Fig 2:**
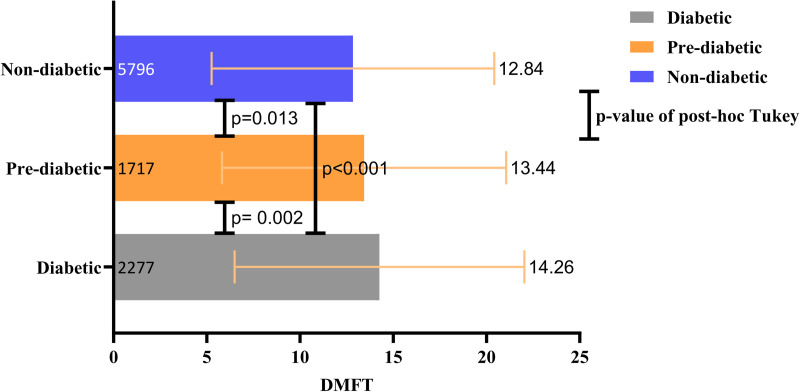
Bar chart indicating mean DMFT ± SD and the results of post-hoc Tukey after eliminating participants with complete dentures.

The mean and standard deviation (SD) of DMFT for all participants were 14.57 ± 8.78. Respectively, 24.05%, 17.47%, and 58.47% of all 10515 subjects were diabetic, pre-diabetic, and non-diabetic. The mean DMFT values in diabetic, pre-diabetic, and non-diabetic individuals were 16.03, 14.63, and 13.94, respectively. The results of the ANOVA analysis revealed a statistically significant difference between the mean DMFT values of diabetic, pre-diabetic, and non-diabetic individuals. To evaluate pairwise differences between the groups, we conducted Tukey’s post hoc analysis, which revealed statistically significant disparities between diabetic and pre-diabetic individuals (p < 0.001), pre-diabetic and non-diabetic individuals (p = 0.009), and diabetic and non-diabetic individuals (p < 0.001). (Fig 1) Also, after eliminating edentulous participants who had complete dentures, the mean DMFT values in diabetic, pre-diabetic, and non-diabetic individuals were 14.26, 13.44, 12.84, respectively. The results of the ANOVA analysis revealed a statistically significant difference between the mean DMFT values of diabetic, pre-diabetic, and non-diabetic individuals. To evaluate pairwise differences between the groups, we conducted Tukey’s post hoc analysis, which revealed statistically significant disparities between diabetic and pre-diabetic individuals (p = 0.002), pre-diabetic and non-diabetic individuals (p = 0.013), and diabetic and non-diabetic individuals (p < 0.001). (Fig 2)

Power analysis confirmed the study’s robustness in detecting group differences in cognitive function. For the overall ANOVA comparing three glucose tolerance groups (Diabetic, Pre-diabetic, Non-diabetic), the achieved power exceeded 99.9% (Cohen’s *f* = 0.098; *N* = 10,520; α = 0.05), indicating near-certain detection of significant between-group variance. In post-hoc pairwise comparisons (Tukey HSD), power remained exceptionally high for Diabetic vs. Non-diabetic (>99.9%; Cohen’s *d* = 0.238; mean difference = 2.09) and Diabetic vs. Pre-diabetic (99.8%; Cohen’s *d* = 0.160; mean difference = 1.40). The comparison between Pre-diabetic and Non-diabetic groups yielded moderate power (87.1%; Cohen’s *d* = 0.078; mean difference = 0.69), suggesting adequate sensitivity to detect even marginal effects in this subgroup contrast. (See Table 2)

**Table 2:** Power analysis results for overall ANOVA and post-hoc pairwise comparisons (Tukey HSD) of cognitive function scores across glucose tolerance groups. The overall ANOVA included three groups (Diabetic, Pre-diabetic, Non-diabetic) with a total sample size of 10,520. Effect sizes are reported as Cohen’s *f* (overall) and Cohen’s *d* (pairwise). All tests used α = 0.05. Power values >99.9% indicate near-complete detection capability.

Analysis Type	Group Comparison	Effect Size	Mean Difference	Sample Size (N)	α	Power (1-β)
Overall ANOVA (3 groups)	—	Cohen’s *f* = 0.098	—	10,520	0.05	**>99.9%**
Pairwise (Tukey HSD)	Diabetic vs. Non-diabetic	Cohen’s *d* = 0.238	2.09	—	0.05	**>99.9%**
Pairwise (Tukey HSD)	Diabetic vs. Pre-diabetic	Cohen’s *d* = 0.160	1.40	—	0.05	**99.8%**
Pairwise (Tukey HSD)	Pre-diabetic vs. Non-diabetic	Cohen’s *d* = 0.078	0.69	—	0.05	**87.1%**

Table 3 presents the results of the ANOVA analysis comparing gender, age, level of education, BMI, and co-morbid diseases among diabetic, pre-diabetic, and non-diabetic groups.

**Table 3:** Comparison of DMFT in diabetic, pre-diabetic, and non-diabetic people based on age, gender, level of education, BMI, co-morbidity diseases, and socioeconomic status.

Variables	Classifications	Diabetic		Pre-diabetic		Non-diabetic		P-Value
		*N*	*DMFT* *Mean ± SD*	*N*	*DMFT* *Mean ± SD*	*N*	*DMFT* *Mean ± SD*	
Age (years)	35-45	442	12.15 ± 8.21	477	10.71 ± 7.15	2219	10.39 ± 6.75	<0.001*
	45-55	862	13.82 ± 7.96	682	13.57 ± 7.61	2308	13.97 ± 7.97	**0.492**
	>55	1226	18.99 ± 9.17	676	18.47 ± 9.09	1623	18.77 ± 9.35	**0.512**
	***P-Value***	<0.001*		<0.001*		<0.001*		
Gender	Male	989	16.82 ± 9.39	907	15.69 ± 8.89	2990	15.36 ± 9.07	<0.001*
	Female	1541	15.52 ± 8.85	928	13.59 ± 8.31	3160	12.60 ± 7.91	<0.001*
	***P-Value***	<0.001*		<0.001*		<0.001*		
Level of Education	illiterate	635	19.90 ± 9.42	319	19.89 ± 9.14	783	20.18 ± 9.29	**0.822**
	Primary	808	15.72 ± 9.06	598	14.01 ± 8.46	1904	13.88 ± 8.54	<0.001*
	Secondary	974	13.93 ± 8.21	814	13.31 ± 7.97	3042	12.62 ± 7.92	<0.001*
	Higher	113	14.64 ± 7.77	104	12.44 ± 7.47	421	12.20 ± 7.14	0.007**
	***P-Value***	<0.001*		<0.001*		<0.001*		
BMI***	<18.5	22	24.40 ± 8.54	20	15.95 ± 10.22	99	19.56 ± 10.67	0.029**
	≥18.5 ≤ 25	546	17.93 ± 9.79	422	17.14 ± 9.09	1777	16.21 ± 9.14	<0.001*
	>25 < 30	1020	16.31 ± 9.14	728	14.66 ± 8.64	2447	13.40 ± 8.42	<0.001*
	>30	942	14.44 ± 8.25	665	12.96 ± 7.95	1827	12.17 ± 7.53	<0.001*
	***P-Value***	<0.001*		<0.001*		<0.001*		
Co-morbidity diseases	No diseases	1562	15.23 ± 8.82	1266	14.40 ± 8.58	4325	13.51 ± 8.37	<0.001*
	One	628	17.28 ± 9.41	407	15.16 ± 8.92	1337	14.73 ± 9.02	<0.001*
	Two or more	340	17.41 ± 9.31	162	15.11 ± 8.61	487	15.68 ± 9.12	0.007**
	***P-Value***	<0.001*		**0.234**		<0.001*		
Physical activity	Low	1034	16.51 ± 8.95	610	14.80 ± 8.78	1857	13.97 ± 8.55	<0.001*
	Moderate	842	15.69 ± 8.96	615	14.16 ± 8.46	2046	13.29 ± 8.31	<0.001*
	High	654	15.71 ± 9.44	610	14.94 ± 8.73	2247	14.52 ± 8.87	0.011**
	***P-Value***	**0.089**		**0.243**		<0.001*		
Socio-economic status	Low	749	14.40 ± 8.09	596	13.84 ± 7.97	2150	12.90 ± 7.76	<0.001*
	Moderate	884	16.60 ± 9.36	594	14.63 ± 8.70	2027	14.03 ± 8.58	<0.001*
	High	897	16.83 ± 9.43	645	15.37 ± 9.17	1973	15.00 ± 9.35	<0.001*
	***P-Value***	<0.001*		0.008**		<0.001*		

*Statistically significant less than 0.001

**Statistically significant less than 0.05

***Body mass index

The results of the ANOVA analysis of smoking habits, using alcohol, using hookah, and using drugs are shown in Table 4. Based on the Pearson correlation, the correlation between the number of cigarettes per day and DMFT is also shown in Table 4.

**Table 4:** comparison of DMFT in diabetic, pre-diabetic, and non-diabetic people based on smoking habits, using alcohol, using hookah, using drugs, and the correlation between the number of cigarettes per day and DMFT.

Variables	Classifications	Diabetic		Pre-diabetic		Non-diabetic		P-Value
		*N*	*DMFT* *Mean ± SD*	*N*	*DMFT* *Mean ± SD*	*N*	*DMFT* *Mean ± SD*	
Smoking habits	Yes	548	19.31 ± 9.51	456	18.61 ± 9.29	1580	17.95 ± 9.35	0.011**
	No	1982	15.12 ± 8.76	1379	13.31 ± 8.02	4570	12.56 ± 7.87	<0.001*
	***P-Value***	<0.001*		<0.001*		<0.001*		
Using alcohol	Yes	332	20.19 ± 9.51	249	19.00 ± 9.77	814	19.96 ± 9.25	**0.271**
	No	2198	15.40 ± 8.86	1585	13.94 ± 8.27	5336	13.03 ± 8.12	<0.001*
	***P-Value***	<0.001*		<0.001*		<0.001*		
Using hookah	Yes	268	17.36 ± 9.54	273	15.23 ± 8.85	974	15.86 ± 9.12	0.018**
	No	2262	15.87 ± 9.02	1561	14.52 ± 8.63	5176	13.58 ± 8.46	<0.001*
	***P-Value***	0.012**		**0.213**		<0.001*		
Using drugs	Yes	136	15.08 ± 9.12	123	13.90 ± 8.88	467	14.50 ± 8.92	**0.568**
	No	2394	16.08 ± 9.09	1711	14.68 ± 8.65	5683	13.90 ± 8.58	<0.001*
	***P-Value***	**0.212**		**0.332**		**0.145**		
Number of cigarettes	**Pearson Correlation**	**0.088**		**0.019**		**0.019**		
	***P-Value***	0.047**		**0.678**		**0.443**		

*Statistically significant less than 0.001

**Statistically significant less than 0.05

ANOVA analyses of brushing habits, flossing, and using mouthwash were conducted and illustrated in Table 5.

**Table 5:** Comparison of DMFT in diabetic, pre-diabetic, and non-diabetic people based on brushing habits, flossing, and using mouthwash.

Variables	Classifications	Diabetic		Pre-diabetic		Non-diabetic		P-Value
		*N*	*DMFT* *Mean ± SD*	*N*	*DMFT* *Mean ± SD*	*N*	*DMFT* *Mean ± SD*	
Brushing habits	Once a day	1149	13.35 ± 7.46	832	12.61 ± 7.13	3009	12.23 ± 7.13	<0.001*
	Twice a day	411	13.07 ± 7.44	317	11.80 ± 7.00	1041	11.16 ± 6.83	<0.001*
	Three times a day	111	13.16 ± 7.93	90	12.47 ± 7.08	283	10.79 ± 7.14	0.008**
	No brushing	272	24.15 ± 8.28	184	23.31 ± 8.41	573	23.34 ± 8.86	**0.411**
	Others	421	15.51 ± 7.58	334	14.10 ± 7.51	1045	13.96 ± 7.58	<0.001*
	***P-Value***	<0.001*		<0.001*		<0.001*		
Flossing	Yes	262	10.80 ± 5.81	246	11.12 ± 5.34	972	10.35 ± 5.75	**0.129**
	No	2268	16.63 ± 9.21	1589	15.17 ± 8.95	5178	14.62 ± 8.88	<0.001*
	***P-Value***	<0.001*		<0.001*		<0.001*		
Using mouthwash	Yes	64	16.85 ± 9.43	60	13.10 ± 7.99	162	13.63 ± 8.70	0.024**
	No	2466	16.01 ± 9.08	1775	14.68 ± 8.68	5988	13.95 ± 8.60	<0.001*
	***P-Value***	**0.463**		**0.163**		**0.638**		

*Statistically significant, less than 0.001

**Statistically significant, less than 0.05

**Fig 3:**
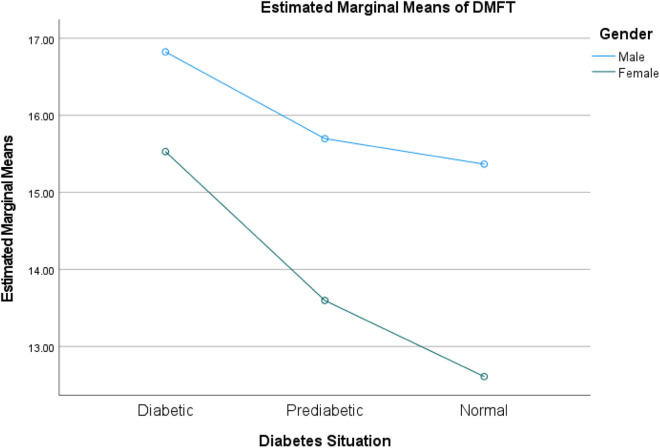
Interaction between diabetic status and gender on DMFT scores. Profile plot showing the significant interaction (p = 0.002) between diabetic status and gender. The varying slopes reveal that gender-based differences in DMFT depend on diabetic status, with all main effects being significant (p < 0.001).

**Fig 4:**
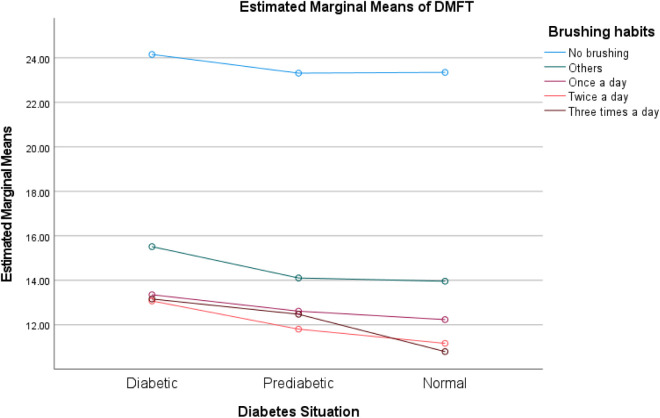
Interaction between diabetic status and brushing frequency on DMFT scores. Profile plot illustrating the relationship between diabetic status (diabetic/prediabetic/normal) and brushing frequency. The nonsignificant interaction (p = 0.512) indicates consistent effects of brushing frequency across all diabetic groups, though both factors independently influence DMFT (p < 0.001 for both main effects).

**Fig 5:**
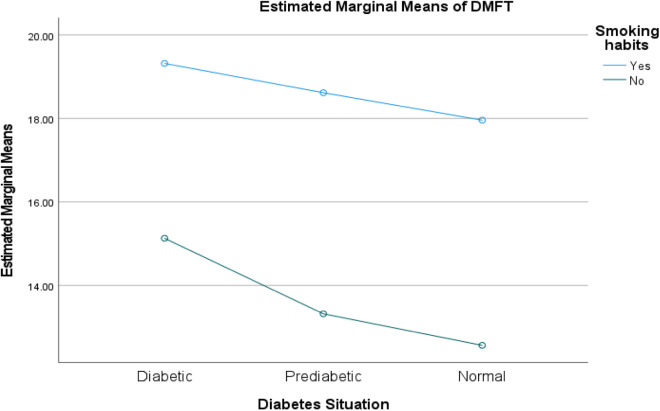
Interaction between diabetic status and smoking status on DMFT scores. Profile plot demonstrating the significant interaction (p = 0.037) between diabetic status and smoking. The non-parallel lines indicate that smoking’s impact on DMFT varies by diabetic group, with both main effects remaining significant (p < 0.001).

The two-way ANOVA analysis reveals that **diabetic status** (diabetic, prediabetic, normal) exerts a statistically significant main effect on DMFT scores across all models (p < 0.001), indicating its strong independent association with dental health outcomes. While **brushing frequency** significantly influences DMFT independently (p < 0.001), its interaction with diabetic status is nonsignificant (p = 0.512), suggesting uniform effects across diabetic groups. Conversely, **smoking status** and **gender** not only demonstrate significant main effects (p < 0.001) but also exhibit significant interactions with diabetic status (p = 0.037 and p = 0.002, respectively), implying that their impact on DMFT varies meaningfully depending on an individual’s diabetic condition. These findings highlight the intricate interplay between diabetic status, behavioral factors (such as smoking and brushing), and demographic variables (gender) in influencing dental health. (See Table 6 and Figs 3–5)

**Table 6:** Results of two-way ANOVA analyses examining the effects of diabetic status (diabetic, prediabetic, normal) and behavioral/demographic factors (brushing frequency, smoking status, gender) on DMFT (Decayed, Missing, and Filled Teeth) scores.

Factor 2	Effect Type	F Statistic	df	p-value	Interpretation
*Brushing Frequency*	Diabetic Status (Main)	F = 21.22	(2,10057)	< 0.001*	Significant independent effect across all brushing levels
	Brushing Frequency (Main)	F = 408.23	(4,10057)	< 0.001*	Strong independent effect on DMFT
	Interaction	F = 0.90	(8,10057)	0.512	Brushing effects are consistent across diabetic groups
*Smoking Status*	Diabetic Status (Main)	F = 34.09	(2,10509)	< 0.001*	Significant independent effect across smoking statuses
	Smoking Status (Main)	F = 507.82	(1,10509)	< 0.001*	Smoking independently worsens DMFT
	Interaction	F = 3.30	(2,10509)	0.037**	Smoking effects differ by diabetic status (significant moderation)
*Gender*	Diabetic Status (Main)	F = 55.31	(2,10509)	< 0.001*	Significant independent effect across genders
	Gender (Main)	F = 112.43	(1,10509)	< 0.001*	Gender independently influences DMFT
	Interaction	F = 6.33	(2,10509)	0.002**	Gender effects vary by diabetic status (significant moderation)

*Statistically significant, less than 0.001

**Statistically significant, less than 0.05

Multiple linear regression was used to adjust for the confounding variables, as illustrated in Table 7.

**Table 7:** Multiple linear regression of DMFT-related factors in diabetic, pre-diabetic, and non-diabetic people.

Variables	Diabetic		Pre-diabetic		Non-diabetic	
	*β Coefficient*	*P-value*	*β Coefficient*	*P-value*	*β Coefficient*	*P-value*
***Age***	0.253	<0.001*	0.274	<0.001*	0.301	<0.001*
***Level of education***	−0.280	<0.001*	−0.292	<0.001*	−0.234	<0.001*
***BMI***	−0.208	<0.001*	−0.193	<0.001*	−0.204	<0.001*
***Flossing***	−2.401	<0.001*	−0.860	**0.110**	−1.459	<0.001*
***Physical activity***	−0.090	<0.001*	−0.62	0.003**	−0.037	0.01**
***Smoking habits***	-.2.195	<0.001*	−3.755	<0.001*	−2.283	<0.001*
***Using alcohol***	1.974	<0.001*	1.722	0.002**	3.302	<0.001*
***Brushing habits***	−2.586	<0.001*	−2.035	<0.001*	−1.947	<0.001*
***Using drugs***	−1.462	0.045**	−0.197	**0.790**	−0.165	**0.656**
***Co-morbidity disease***	0.393	**0.073**	−0.296	**0.279**	0.308	0.040**
***Using mouthwash***	1.722	**0.082**	0.978	**0.320**	0.386	**0.505**
***Socioeconomic status***	−0.286	**0.216**	−0.414	**0.099**	−0.542	<0.001*
***Gender***	0.466	**0.318**	0.334	**0.485**	0.364	**0.166**
***Using hookah***	−0.106	**0.857**	−1.504	0.010**	−0.516	**0.091**
***Residency***	−0.36	**0.912**	0.458	**0.215**	0.313	**0.112**

*Statistically significant less than 0.001

**Statistically significant less than 0.05

The results showed that in diabetic participants, older age, lower levels of education, lower BMI, not flossing, less physical activity, smoking, using alcohol, brushing teeth less, and using drugs were risk factors in increasing the DMFT index. The results showed that in pre-diabetic participants, older age, lower levels of education, lower BMI, less physical activity, smoking, using alcohol, brushing teeth less, and using hookah were risk factors in increasing the DMFT index. The results showed that in non-diabetic participants, older age, lower levels of education, lower BMI, not flossing, less physical activity, smoking, using alcohol, brushing teeth less, having other diseases, and lower socioeconomic status were risk factors in increasing the DMFT index.

## Discussion

DM is a persistent metabolic and heterogeneous disease characterized by hyperglycemia [1,24]. Chronic hyperglycemia and related metabolic dysregulation can lead to damage to various organs, particularly the nerves, kidneys, eyes, and blood vessels. Hyperglycemia also leads to oral complications such as gingivitis, periodontal diseases, salivary dysfunction, tooth caries, altered taste, xerostomia, halitosis, poor wound healing, oral mucosal diseases, and infections such as lichen planus, oral candidiasis, and recurrent aphthous stomatitis [25–29].

In our study, the mean DMFT in the diabetic, pre-diabetic, and non-diabetic groups was 16.03, 14.63, and 13.94, which indicates that oral health is more compromised in participants with diabetes compared to those in a pre-diabetic state, and in pre-diabetic participants, it is more adversely affected than in those with a healthy state. Other studies were consistent with ours [12,30]. Saliva is a crucial factor in maintaining oral health with its various components, such as calcium, immunoglobulins, alkaline phosphates, inorganic phosphorus, and proteins. Alterations in the composition of saliva, including changes in buffering capacity, viscosity, flow rate, and PH, can impact the microbial load, leading to demineralization of enamel and the onset of caries [25]. Hyperglycemia causes increased advanced glycosylation end products, known as “AGEs”. Crosslinking of proteins such as collagen and extracellular matrix proteins occurs due to the formation of advanced glycation end-products (AGEs). The crosslinks cause changes in the basement membrane that lead to endothelial dysfunction. As a result, the microvasculature structure alters and becomes more permeable. Chronic hyperglycemia has been observed to bring about changes to the basement membrane through the generation of sorbitol, diacylglycerol, and fructose-6-phosphate, leading to the alteration of the matrix proteins. In the end, microvasculature and basement leakage are achieved, which causes increased glucose passage into saliva and the crevicular space [27,28]. As a result, decreased activity of fibroblasts is observed, leading to increased plaque formation [25,27]. Endogenous bacteria such as Streptococcus sobrinus, Streptococcus mutans, and Lactobacillus species in the plaque biofilm produce organic acids by fermenting dietary carbohydrates. Elevated glucose levels in saliva increase Streptococcus mutans fermentation activity on dental plaque, which lowers saliva pH and promotes the growth of cariogenic and acidophilic bacteria [25,28,31–34]. As per the information shared earlier, enamel demineralization is expedited, leading to an increased occurrence of caries [27]. As was explained above, diabetes increases the caries rate; therefore, elevated DMFT due to diabetes is expected.

The present study revealed that participants with a lower BMI in two groups- diabetic and non-diabetic – have worse oral health than participants with a higher BMI. Some of the previous studies revealed the same outcome as the present study [35–38]. The underweight participants have the possibility of malnutrition or undernutrition [39]. Malnutrition and undernutrition can influence the maturation of teeth, which leads to hypomineralized enamel or poorly developed enamel. Consequently, the likelihood of experiencing decay rises. Malnutrition also causes a reduction in salivary function through protein-energy malnutrition, which leads to a decrease in the secretion of protein, a decrease in the flow rate of saliva, and a decrease in buffering capacity [40]. A decrease in the function of saliva can increase the risk of decay by increasing tooth enamel solubility [41,42]. As a result, the incidence of caries rises [43,44]. Furthermore, dental caries and periodontitis can cause tooth loss, resulting in inappropriate biting or chewing of food. As a consequence, losing weight is expected [45]. In contrast with the last sentence, some studies revealed the opposite outcome [46–48]. Globally, obesity has increased over the last few years [49]. Obesity is associated with many systemic diseases, but obesity’s effect on oral health is a subject of controversy [50]. Diet stands out as a significant etiological factor for both dental caries and obesity. The correlation between dental caries and obesity is significantly influenced by a diet rich in refined sugars and carbohydrates [51]. Many other pathways may exist. For example, the concept of ‘infectobesity’ assumes that obesity may arise from an infectious source [52,53]. Hence, high levels of Firmicutes in the gut microflora have been linked to obesity [54]. Similar bacterial species are observed in both the gut and dental microflora. Insulin resistance and inflammation are factors believed to play a role in this process [55,56]. In obesity, hyperplasia and hypertrophy of adipocytes cause low-grade, chronic systemic inflammation. When it comes to obesity, adipocytes exhibit an elevation in the production of proinflammatory cytokines, including tumor necrosis factor-alpha, interleukin 6, and interleukin 1. Tumor necrosis factor-alpha is among the first proinflammatory cytokines released in periodontal disease. It causes the initiation of periodontal disease by stimulating the formation of osteoclasts and the destruction of alveolar bone [57,58]. Therefore, tooth loss is more likely due to periodontal disease, resulting in a higher DMFT in obese participants compared to those who are underweight.

Our data shows that smoking and using hookah have effects on the oral health of all 3 groups and diabetic and non-diabetic participants, respectively. Similar findings were observed in another study [12]. The interaction observed in Table 5 suggests that smoking may have differential effects on DMFT scores depending on diabetic status, potentially reflecting varying physiological responses to tobacco exposure across different metabolic states. This finding is particularly relevant given that smoking prevalence remains high among diabetic populations despite increased health risks [59,60]. Smoking has negative effects on the oral cavity by causing adverse chemical, mechanical, and physical side effects. Tobacco smoke changes the salivary flow rate, PH, and the oral commensals [61–63]. Long-term consumption of tobacco decreases saliva secretion (sialopenia) and dry mouth by decreasing the response of taste receptors and affecting the gustatory reflex. Salivary buffering capacity is also reduced by approximately 20% in smokers compared to nonsmokers, which leads to acidic PH [64,65]. It is found that tobacco smoke increases caries-promoting microbiota such as Lactobacilli in saliva, which may result from two reasons: first, smokers have more carious teeth than nonsmokers, and second, authors suggest that smoking depresses the immunoglobulins, such as IgM and IgA, in the oral cavity, which leads to a higher count of bacteria [66]. In addition, nicotine can enhance cariogenic microorganisms such as Streptococcus mutans, Actinomyces, Lactobacilli, Candida albicans, and Streptococcus gordonii by creating a caries-susceptible environment. Moreover, nicotine can decrease the competitive capability of commensal microorganisms such as Streptococcus sanguinis [67].

Using alcohol elevated the DMFT index and impaired the state of oral hygiene in all three groups of diabetics, prediabetics, and healthy adults in the present research. Our research aligns with the outcomes reported in other studies [68–70]. Alcohol consumption can lead to a higher DMFT index and worse oral health in three different ways. Alcoholics are known to have inadequate oral hygiene and professional and personal dental care. High consumption of refined carbohydrates and the experience of dry mouth and vomiting may be associated with a higher DMFT index among them [68]. In addition, alcoholic drinks promote tooth wear directly. High levels of polyphenols, mostly tannins, present in alcoholic beverages bind salivary proline-rich proteins and precipitate them, which causes the astringency and typical taste of some alcoholic drinks and loss of lubrication of saliva and teeth, which leads to less protection of teeth from acids [71]. Most alcoholic drinks have an acidic PH, which is around PH 4.0, which is related to the high level of organic and inorganic acids present in alcoholic drinks. In addition, the habit of keeping alcoholic beverages in the mouth is an important factor in developing tooth wear [71–73]. Alcohol abuse can increase the rate of chronic periodontal inflammation, gingivitis, interdental papillae bleeding, and deep gingival pockets, which are related to bone loss [74,75]. Alcoholic drinks can affect periodontal tissues indirectly due to adverse effects on the immune system. It can cause complement deficiency and impaired neutrophil function (by decreasing adherence, motility, and phagocytic activity), which can lead to frequent infection of periodontal tissue. In addition, alcohol can disrupt clotting mechanisms, vitamin K activity, and prothrombin production due to its toxic effects on the liver, which can result in gingival inflammation and bleeding [76].

Our study shows that brushing improves oral and dental hygiene and also causes less DMFT. Other studies were consistent with ours [77–80]. In our study, there are contradictions related to the lowest DMFT in 3 diabetic, pre-diabetic, and healthy groups, according to the number of brushing times per day, and we believe that this contradiction is due to the difference in brushing methods among people. Our study indicates that flossing improves oral hygiene and reduces DMFT. Other studies also confirmed our study [79–82]. Dental plaque plays a pivotal role in developing gingival and periodontal diseases [83,84]. People have been using different techniques to manage their oral hygiene since ancient times [84]. Flossing and regular tooth brushing are key oral hygiene practices recommended by dentists for good oral health [83,85,86]. Brushing teeth removes food particles. Therefore, it can modify the effect of daily intake on dental caries. Toothbrushing also regulates the cariogenic bacteria count [78]. For interproximal and subgingival regions that a toothbrush can’t clean, flossing prevents caries by scrubbing food particles off [85,87,88]. Flossing has the greatest benefit for molars because molars are the most commonly missing teeth, because they are inaccessible and have a multi-root nature [81].

Unlike smoking and gender, brushing frequency showed no significant interaction with diabetic status (p = 0.512), despite having a strong main effect (p < 0.001). This finding suggests that good oral hygiene practices, particularly regular tooth brushing, provide consistent benefits across all diabetic groups. This observation aligns with extensive research demonstrating that improved oral hygiene behaviors are associated with better oral health outcomes regardless of diabetic status [89–92].

The lack of interaction for brushing frequency indicates that oral hygiene education and promotion should be universally beneficial across diabetic, pre-diabetic, and non-diabetic populations. This is particularly important for clinical practice, as it suggests that standard oral hygiene recommendations remain effective across different metabolic states [93–95].

However, this study has limitations.

- Oral examinations were conducted by two educated, skilled examiners. This problem was solved by training and supervising by a dentist, following WHO guidelines, before performing the study.
- The amount of alcohol consumption or the type and administration route of drugs is not available or discussed.
- While DMFT is a validated measure of dental caries, it does not evaluate periodontal health, oral hygiene, or quality of life. Thus, our findings should be interpreted as specific to caries experience rather than overall oral health. Finally, although sensitivity analyses excluding edentulous participants confirmed the robustness of our primary results, future studies should incorporate multidimensional oral health assessments.

## Conclusion

In all groups, higher DMFT risk factors included older age, lower education, reduced BMI, less physical activity, smoking, alcohol consumption, and inadequate teeth brushing. Notably, drug use is regarded as a risk factor exclusively among participants with diabetes.

## Supporting information

S1 File STROBE checklist-v4 combined. (DOCX)
